# P-49. Sexual and Gender Minority Veterans With HIV More Likely Vaccinated Against HPV Than Those Without HIV in a Nationwide Cohort

**DOI:** 10.1093/ofid/ofae631.256

**Published:** 2025-01-29

**Authors:** Puja Van Epps, Wyatt E Meriwether, Alexis (Lexi) Matza, Alex Mcconnell, Jillian Shipherd, Michael R Kauth

**Affiliations:** Veterans Health Administration, Case Western Reserve University School of Medicine, Cleveland, Ohio; Veterans Health Administration, Kansas City, Missouri; Veterans Health Administration, Kansas City, Missouri; Veterans Health Administration, Kansas City, Missouri; Veterans Health Administration/ Boston University, Boston, Massachusetts; Veterans Health Administration/University of Massachusetts, Worcester, Massachusetts

## Abstract

**Background:**

Certain sexual and gender minority (SGM) adults, particularly those with HIV, experience a disproportionate impact of Human Papillomavirus (HPV) and its related diseases. Vaccination against HPV, which is recommended in all adults up to age 26 and in some up to age 45, is an important tool in addressing this disparity. In non-Veteran cohorts, young SGM adults, particularly of non-White race are less likely to be vaccinated against HPV. We examined HPV vaccination rates in the Veterans Health Administration (VHA) among a nationwide cohort of age eligible SGM Veterans and report on disparities across racial groups and HIV status

**Figure:**
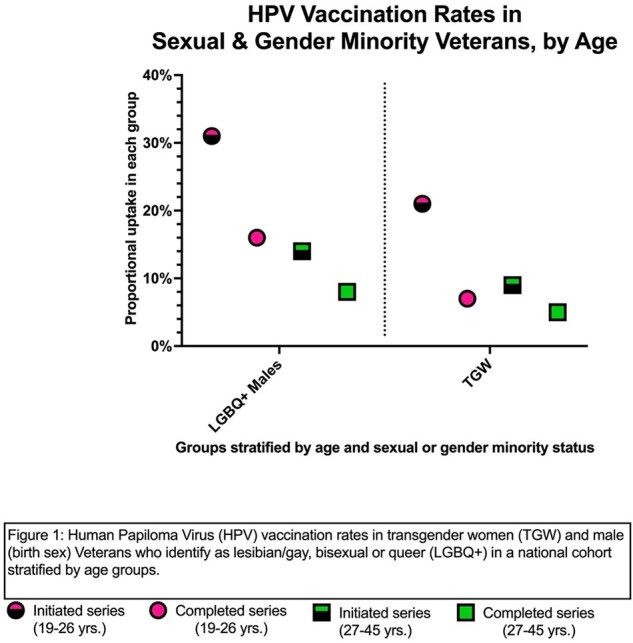

**Methods:**

We queried VHA’s national database to identify Veterans whose sexual orientation is recorded as lesbian, gay, bisexual or queer (LGBQ+) and gender identity as transgender woman (TGW) as of April 2024. Birth sex male LGBQ+ and TGW Veterans of both birth sexes were included in the cohort, stratified by age, race/ethnicity and HIV status. Within these cohorts we quantified receipt of HPV vaccine doses in the VHA starting 2022 and compared rates of vaccine initiation or completion across groups.

**Figure:**
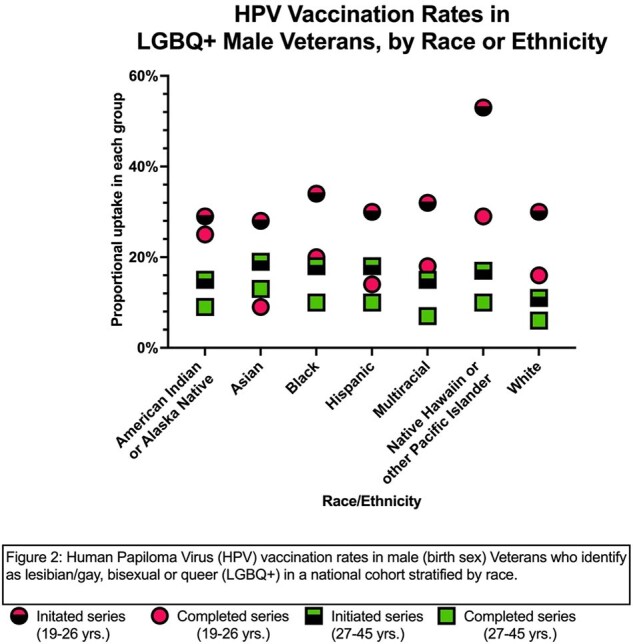

**Results:**

Among the 1802 LGBQ+ males age 26 and under, 561 (31%) had received at least one dose of the HPV vaccine in the VHA; of these, 50% had completed the series (Figure 1). In the 27-45 age group, 2082/14863 (14%) had initiated and of these, 60% had completed the series. Non-Whites in both age groups had higher rates of series initiation compared with Whites (19-26: 34% vs. 30%; 27-45: 17% vs. 11%) (Figure 2). Those with HIV had higher rates of series initiation in both age groups compared to those without (19-26: 52% vs. 30%; 27-45: 26% vs. 16%) (Figure 3). Among TGW: 26 and under (n=154), 21% had received at least one dose and 38% of these have completed the series; 27-45 (n=932) 9% had initiated the series, but rates were much higher in those with HIV in this group (series initiation: 33%; completion rate: 63%).

**Figure:**
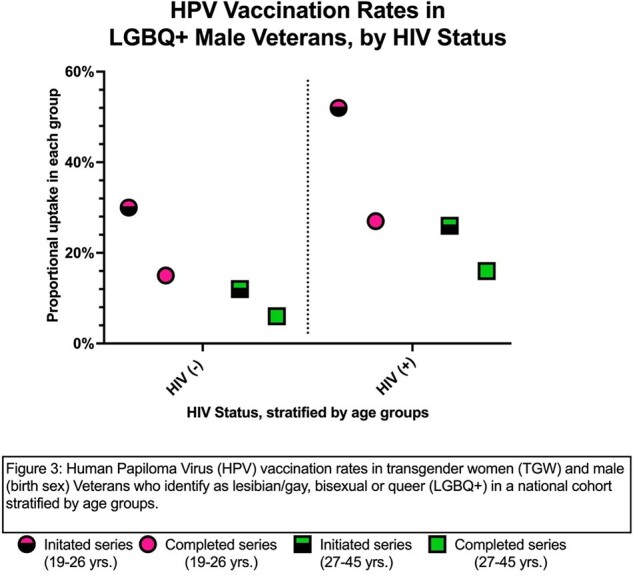

**Conclusion:**

Less than a third of eligible SGM Veterans have received HPV vaccination in the VHA. Unlike non-Veteran cohorts, younger age and non-White race were associated with increased vaccination rates, with rates among those with HIV being the highest and those among TGW the lowest. More efforts are needed to increase HPV vaccination in these high need populations.

**Disclosures:**

**All Authors**: No reported disclosures

